# The efficacy and safety of auricular point pressing therapy for knee osteoarthritis: A protocol for systematic review and meta-analysis

**DOI:** 10.1097/MD.0000000000029098

**Published:** 2022-04-15

**Authors:** Jie He, Lin Ma, Feng Zhou, Hongbo Jiang, Huajie Wang, Xin Wang, Yuxin Zhao

**Affiliations:** Jiangyan Hospital Affiliated to Nanjing University of Chinese Medicine, Taizhou, Jiangsu Province, China.

**Keywords:** auricular point pressing therapy, knee osteoarthritis, meta-analysis, protocol, Western Ontario and McMaster Universities Osteoarthritis Index

## Abstract

**Background:**

Knee osteoarthritis (KOA) is one of the leading causes of disability. The effectiveness of auricular point pressing therapy for treating KOA remains controversial. This protocol describes the method of a systematic review and meta-analysis evaluating the effectiveness and safety of auricular point pressing therapy for treating KOA.

**Methods:**

Four English databases (PubMed, Embase, Cochrane Library databases and Web of Science) and 4 Chinese databases (China National Knowledge Infrastructure, Chinese Biomedical Literature Database, VIP Database for Chinese Technical Periodicals, and Wanfang) will be searched. All randomized controlled trials related to auricular point pressing therapy for KOA will be included. Extracted data will include publication details, basic information, demographic data, intervention details and patient outcomes. The primary outcome will be Western Ontario and McMaster Universities Osteoarthritis Index and visual analogue scale. Risk of bias will be assessed using the Cochrane Collaboration tool for assessing risk of bias. Article selection, data extraction and risk of bias assessment will be performed by 2 independent reviewers. If the meta-analysis is precluded, we will conduct a descriptive synthesis using a best-evidence synthesis approach. The strength of recommendations and quality of evidence will be assessed using the Grading of Recommendations Assessment Development.

**Results:**

The systematic review will provide evidence to assess the efficacy and safety of auricular point pressing therapy on KOA.

**Conclusion:**

The systematic review will provide evidence to assess the efficacy and safety of auricular point pressing therapy for KOA patients.

**Ethics and dissemination:**

For this review, ethical approval is not required. Patients will not be involved. The findings will be published in a peer-reviewed journal.

**INPLASY registration number:**

INPLASY 202220077.

## 1 Introduction

Osteoarthritis (OA) is a common clinical degenerative disease and is one of the leading causes of disability.^[1]^ The common symptoms of OA are pain, stiffness and dysfunction of the joint, which have a serious impact on quality of life.^[2]^ In USA, the costs of OA patients about at 45 billion 1 year.^[3]^ Approximately 85% of global OA burden is knee osteoarthritis (KOA).^[4]^ The main goals of treatment are relieving pain and improving function. In order to achieve this purpose, several options are applied, such as weight loss, exercise, corticosteroid injections, nonsteroidal anti-inflammatory drugs (NSAIDs), and glucosamine suppletion. However, it is difficult exercise and loss weight. NSAIDs are the most commonly used for pain management accompanied with the risk of gastrointestinal bleeding and vascular adverse events.^[5]^ In addition, the joint replacement requirements costs are increasing the burden of healthcare systems of Western countries. In this context, it is important to develop novel therapies that can decrease joint pain and have less adverse effects.

Traditional Chinese medicine (TCM) treatments include variations of nonpharmacologic therapies. Auricular point pressing therapy was one of acupuncture-related techniques used as treatment in China over 2000 years.^[6]^ According to TCM theory, the ear contains acupoints that reflect the whole body.^[7]^ Auricular point pressing therapy is one of the external therapies with the characteristics of TCM, which has been widely used in Chinese hospital to treat pain.^[8,9]^ Several studies result showed that auricular acupuncture had a significant effect on postoperative pain.^[10,11]^ At present, no systematic review reported on the efficacy and safety of auricular point pressing therapy for KOA. Therefore, according to the objective clinical needs, we design this systematic review and meta-analysis of randomized controlled trials (RCTs) on the effect of auricular point pressing therapy on KOA, so as to provide support for the spread of evidence.

## 2 Methods

This systematic review protocol has been registered on INPLASY (https://inplasy.com/inplasy-2022–2–0077/). The registration number is INPLASY 202220077. This protocol was reported to follow the statement guidelines of preferred reporting items for systematic reviews and meta-analysis protocol.^[12]^ Ethical approval is unnecessary because this is a literature-based study.

### 2.1 Inclusion criteria

#### 2.1.1 Type of studies

All RCTs of auricular point pressing therapy for KOA without publication status restriction or writing language will be included, with or without blinding. Non-RCTs, quasi-RCTs, uncontrolled trials, reviews, case-controlled studies, animal trials, and laboratory studies will be excluded.

#### 2.1.2 Types of participants

KOA patients with definite diagnosis will be included. The diagnostic criteria should be based on the American College of Rheumatology clinical criteria, National Institute for Health and Clinical Excellence guidelines or any other accepted guidelines.^[13]^ There will also be no limitations related to age, sex, education, disease duration, and disease severity.

#### 2.1.3 Type of interventions

The experimental group was treated with auricular point pressing therapy. The control group was treated with placebo, drugs, or other alternative therapy.

#### 2.1.4 Types of outcomes

##### 2.1.4.1 Primary outcome

The primary outcome is Western Ontario and McMaster Universities Osteoarthritis Index and visual analogue scale. Western Ontario and McMaster Universities Osteoarthritis Index is a self-report questionnaire for OA of the hip or knee, with higher scores indicating more serious pain, poorer physical function, and increased stiffness.

##### 2.1.4.2 Secondary outcomes

Lequesne index and Medical Outcomes Study Short Form 36 health survey will be accepted as the secondary outcomes.

##### 2.1.4.3 Safety outcomes

The incidence and severity of side effects will be used to evaluate the safety. Any unexpected events occurred will be recorded.

### 2.2 Search methods for identification of studies

#### 2.2.1 Electronic searches

We will develop search strategies for 4 English databases (PubMed, EMBASE, Cochrane Library and Web of Science) and 4 Chinese databases (China Knowledge Resource Integrated Database, Weipu Database for Chinese Technical Periodicals, Sinomed, and Wanfang Database). There is no restriction for language or publication status. The search terms include auricular, point, points, KOA, gonarthrosis, osteoarthrosis, osteoarthropathy, and arthralgia. The detailed strategies for searching the PubMed database are presented in Table 1. The similar search strategies are applied to other electronic databases.

**Table 1:** Search strategy used in PubMed.

Search	Search terms
1	((((knee osteoarthritis) OR gonarthrosis) OR osteoarthrosis) OR osteoarthropathy) OR arthralgia
2	((auricular) OR point) OR points
3	(((random [Text Word] OR randomized [Text Word]) OR control [Text Word]) OR controlment [Text Word]) OR trial [Text Word] AND “humans” [MeSH Terms]
4	#1 AND #2 AND #3

#### 2.2.2 Searching other resource

Additionally, we will search the international clinical trials registry platform, dissertation, and gray literature to identify relevant studies. Conference papers, journals will be retrieved manually.

### 2.3 Data collection and analysis

#### 2.3.1 Selection of studies

**Figure 1:**
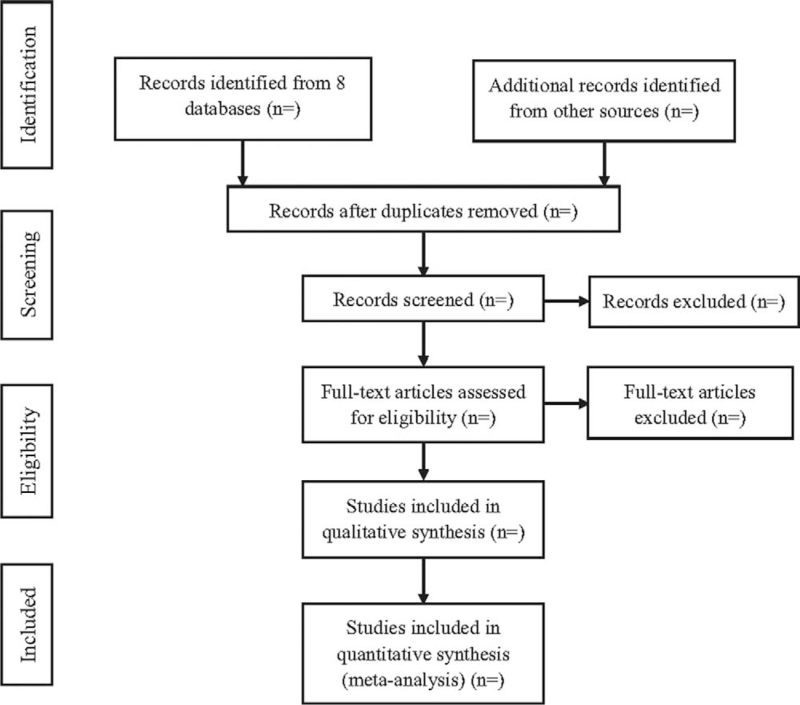
Flow diagram of the study selection process.

All search literatures will be exported to endnote, which will be checked. The duplications will be removed. Two reviewers will screen all titles and abstracts independently to identify potentially relevant studies. The 2 reviewers will read the full text to confirm which studies meet the inclusion criteria. Any disagreement will reach consent by discussion. The reasons of excluding will be recorded. The study selection process is demonstrated in Figure 1.

#### 2.3.2 Data extraction and management

The standard data extraction form will be designed for data collection. Two reviewers will extract all data independently using the predesigned template. Disagreements will be settled by discussion. Two reviewers will cross-check all the data. We will contact the corresponding authors for more information if it is necessary. The extracted study characteristics include the first author, publication year, country, location, study type, sample size, length of follow-up, type of intervention and control, outcome assessment, main conclusions, adverse events.

#### 2.3.3 Assessment and quality of included studies

Two reviewers will assess the methodological quality of included studies with the Cochrane Collaboration tool independently. The quality evaluation items include these items: selection bias (random sequence generation and allocation concealment), performance bias (blinding of researchers and participants), attrition bias (incomplete outcome data), ascertainment bias (blinding of outcome assessment), reporting bias (selective outcome reporting) and other sources of potential bias. According to the criteria, the study will be classed as “low risk of bias”, “high risk of bias”, “unclear risk of bias”. Any disagreement will be solved by discussion.

#### 2.3.4 Management of missing data

For the incomplete data, we will try to contact the first or corresponding author by email if necessary. We will analyze the data acquired when the missing data is not available.

#### 2.3.5 Assessment of heterogeneity

The research will be performed by Review Manager version 5.3 software (The Nordic Cochrane Centre, Copenhagen). *P *< .05 will be defined as statistically significant between studies. Meta-analysis will be performed to identify the effectiveness of auricular point pressing therapy when there are enough data. Heterogeneity among the included will be assessed by *I*^2^ statistic.^[14]^ Summary estimates will be presented in forest plots. When *I*^2^ is less than 50% indicating low heterogeneous. If the *I*^2^ is more than 50%, meta-regression and subgroup analyses will be conducted to explore the possible sources of heterogeneity. If the meta-analysis cannot be performed, a descriptive synthesis will be provided.

#### 2.3.6 Analysis of subgroup

If the meta-analysis shows significant heterogeneity in the studies, subgroup analyses will be performed to explain the heterogeneity. Predefined subgroup includes the location of studies, the type of intervention, the dosage of auricular point pressing, the stage of KOA.

#### 2.3.7 Data synthesis

All data will be combined and analyzed by Cochrane Collaboration software RevMan V.5.2 (The Nordic Cochrane Centre, Copenhagen) and Stata V.12.0 (Version 12.0; Stata Corporation). The random-effect model will be used for all the analyses because the RCTs included may come from different people. For dichotomous variables, the Mantel Haenszel method will be used for analyses. Relative risk with 95% confidence intervals will be reported for effect size. For continuous variables, the inverse variance method will be used for analyses and treatment effect will be reported as mean difference with 95% confidence intervals.

#### 2.3.8 Sensitivity analysis

The sensitivity analysis will be conducted to verify the stability of the review conclusions. We will consider removing 1 study at a time to observe its effect on heterogeneity and effect size.

#### 2.3.9 Assessment of reporting biases

In terms of accuracy, funnel plot will be performed to assess the reporting bias,^[15–17]^ if there are 10 or more studies. The Egger and Begg tests will be performed to assess funnel plot asymmetry. We will define significant publication bias as a *P* value <0.1.

#### 2.3.10 Confidence in cumulative evidence

The quality level of evidence will be analyzed using the grading of recommendations assessment, development, and evaluation system, including 4 levels: high, moderate, low, or very low.^[18]^

#### 2.3.11 Ethics and dissemination

The content of this article does not involve moral approval or ethical review and would be presented in print or at relevant conferences.

## 3 Discussion

This systematic review will be performed based on previous studies of auricular point pressing therapy for KOA. The conclusions from this study may affect KOA patients, clinicians and policy-makers. OA is a major cause of disability in older people. It is a slowly progressive and chronic condition decreasing the quality of life. The process of OA involves many cytokine and grow factors or signaling pathways.^[19–21]^ NSAIDs can significantly relieve KOA pain. However, the adverse effects of drugs should cause clinician to take seriously. Auricular point pressing therapy is commonly used in Chinese hospitals. Points on the ear are stimulated to treat various disorders, especially for patients with pain. The most common symptom of KOA is pain. It is crucial to determine whether auricular point pressing therapy is a benefit choice for KOA. Therefore, the purpose of this proposed systematic review is to evaluate the efficacy and safety of auricular point pressing therapy for KOA. In this review, we provide a systematic review based on the most recent evidence from clinical studies.

## Author contributions

**Conceptualization:** Jie He, Lin Ma.

**Data curation:** Jie He, Feng Zhou, Yuxin Zhao.

**Formal analysis:** Lin Ma, Huajie Wang.

**Funding acquisition:** Huajie Wang, Xin Wang.

**Investigation:** Feng Zhou, Hongbo Jiang Jiang.

**Methodology:** Hongbo Jiang Jiang, Huajie Wang, Xin Wang.

**Project administration:** Lin Ma, Huajie Wang.

**Resources:** Jie He, Lin Ma.

**Software:** Feng Zhou, Huajie Wang, Xin Wang.

**Supervision:** Lin Ma, Xin Wang, Yuxin Zhao.

**Validation:** Hongbo Jiang Jiang, Huajie Wang, Xin Wang.

**Visualization:** Lin Ma, Huajie Wang.

**Writing – original draft:** Jie He, Lin Ma, Yuxin Zhao.

**Writing – review & editing:** Jie He, Feng Zhou.
